# An overview of thyroid eye disease

**DOI:** 10.1186/s40662-014-0009-8

**Published:** 2014-12-10

**Authors:** Colm McAlinden

**Affiliations:** College of Medicine, Swansea University, Swansea, UK

**Keywords:** Thyroid eye disease, Graves’ disease, Graves’ ophthalmopathy, Hyperthyroidism, Hypothyroidism, Euthyroidism

## Abstract

Thyroid eye disease (also known as Graves’ ophthalmopathy) is a complex orbital inflammatory disease, which can be sight threatening, debilitating and disfiguring. This overview discusses the epidemiology, risk factors, pathogenesis, presentation, ophthalmic clinical features, investigations and treatment of thyroid eye disease.

*The beauty of a woman must be seen from her eyes, because that is the doorway to her heart, the place where love resides.*

Audrey Hepburn

## Introduction

Thyroid eye disease (TED) is a complex orbital inflammatory disease, which can be sight threatening, debilitating and disfiguring. TED is also known as Graves’ ophthalmopathy, named after Robert J. Graves, an Irish physician who first described thyrotoxicosis in a woman presenting with goitre, rapid heartbeat and exophthalmos [1].

The acute progression of the disease is an ocular emergency, particularly optic nerve compression and corneal disease secondary to exposure. Most patients with TED have biochemical evidence of hyperthyroidism with the most common cause being Graves’ disease. However, TED may occur in patients who have hypothyroidism (most commonly Hashimoto’s thyroiditis) or euthyroidism. Hence, the timing of TED presentation may differ between patients. There are patients wherein thyroid dysfunction precedes TED development; there are patients in whom thyroid dysfunction and TED present at the same time, and there are patients where TED is the first manifestation, preceding thyroid dysfunction [2,3].

## Review

### Epidemiology

The incidence of TED is 16 per 100,000 females and 2.9 per 100,000 males with an approximate prevalence of 0.25% with no significant ethnic predisposition [4]. The higher preponderance in females relates to the higher incidence of hyperthyroidism in females. However, for severe TED, the ratio of females to males reverses to approximately 1:4 [5].

### Risk factors

Risk factors for the disease include female gender, middle age and smoking [6]. Smoking increases the risk of TED by 7–8 times [2] and it reduces the effectiveness of treatments [7]. There have been reports suggesting that polymorphisms in genes such as human leukocyte antigen (HLA), cytotoxic T-lymphocyte antigen 4 (CTLA4), interleukin 23 receptor (IL23R), protein tyrosine phosphatase nonreceptor 22 (PTPN22), CD40, CD86, thyroglobulin (Tg) and thyroid stimulating hormone receptor (TSHR) increase the risk of TED [8,9]. However, reported associations may vary considerably between different populations and the majority of studies lack adequate sample size and power to detect associations with occurrence and severity of TED. Thus, although variations in genes, especially those encoding immunological factors, have been associated with TED, large and well controlled studies are required to determine the exact contribution of gene variations. A large recent study by Yin et al. concluded that patients with TED do not have a distinct genetic susceptibility to their eye disease and suggested that environmental and/or epigenetic influences are at play [10]. In addition, mechanical factors and orbital anatomy have been suggested to influence the occurrence and clinical course of TED [11].

In autoimmune cases of Graves' disease or Hashimoto's thyroiditis, there is also an increased prevalence and relative risk for coexisting autoimmune disorders [12]. Examples include rheumatoid arthritis, pernicious anaemia, systemic lupus erythematosus, Addison's disease, coeliac disease, and vitiligo. Hence, it is important to screen for other autoimmune diseases if subjects with autoimmune thyroid disease present with new or nonspecific symptoms.

### Pathogenesis

TED is caused by retro-orbital inflammation to which orbital fibroblast activation is a key contributor. Fibroblast activation is presumed to occur secondary to stimulatory auto-antibodies [anti-TSHR and anti-insulin-like growth factor-1 (IGF-1)] [13]. These fibroblasts express the TSH receptor and produce extracellular matrix components and pro-inflammatory molecules. Further, there is an infiltration of immunocompetent T-helper cells (type-1), B lymphocytes, macrophages and mast cells [14]. Inflammation of the extraocular muscles can lead to restricted eye movements and proptosis. The optic nerve can be compressed which can cause optic neuropathy resulting in permanent visual loss.

However, there are situations when TSHR antibodies are not present, such as in cases of Hashimoto’s thyroiditis and eye disease and euthyroid Graves’ disease [15]. In addition, autoimmunity against the eye muscle antigen calsequestrin and the orbital connective tissue antigen collagen XIII plays a role in the pathogenesis of TED [15-17].

A unique feature of TED in comparison to other autoimmune diseases is that it is self-limiting. The suggested reason is the absence of lymphoid tissue (and hence, lymphoid neogenesis) within the orbit [18]. The disease commences with an active (inflammatory) phase with rapidly worsening symptoms and signs, reaching a point of maximum severity which then improves to a static plateau but does not resolve to baseline (inactive phase). This is known as Rundle’s curve [19] and can be plotted graphically for each patient but is rarely performed in practice. Rather, precise clinical documentation of the severity and activity of the disease is usually preferred.

### Presentation

In approximately 40% of patients with TED, the ocular and systemic symptoms have a simultaneous onset [20]. Approximately 60% of patients with hyperthyroidism will develop TED. For those with TED, 85% have hyperthyroidism, 10% have hypothyroidism and 5% are euthyroid [21].

### Ophthalmic clinical features

Symptoms include: dry eyes, red eyes, diplopia, pain on eye movement and cosmetic changes. Signs include: proptosis (exophthalmos), lid retraction, lid lag on downgaze, chemosis, conjunctival injection, orbital fat prolapse, keratopathy, periorbital swelling, restrictive myopathy and optic neuropathy [22]. However, the most common clinical sign is eyelid retraction (occurs in 90% of patients with TED), followed by exophthalmos (60%) and eye movement restrictions (40%) [23].

The two most serious signs are optic neuropathy and exposure keratopathy as both can abruptly lead to blindness and are therefore ocular emergencies. There are a number of grading systems available in an attempt to document the severity and activity of the disease such as the NOSPECS classification [24] and Mourits system [25]. However, such classifications tend only to be used for research purposes. Other grading systems include The European Group on Graves’ Orbitopathy (EUGOGO) protocol for assessment of Graves’ orbitopathy [26] and the VISA classification (V – vision, optic neuropathy; I – inflammation, congestion; S – strabismus, motility restriction; A – appearance, exposure).

### Investigations

Investigations include thyroid function tests [TSH, free T4 and free T3 (if strong clinical suspicion but normal TSH and free T4)], thyroid auto-antibodies (anti-TSH receptor, anti-thyroid peroxidase and anti-Tg antibodies), orbital imaging [magnetic resonance imaging (MRI) is better for identifying active disease (muscle bellies show inflammation and enlargement with spared tendons) whereas computed tomography (CT) is better for bone resolution when planning decompression surgery], visual field analysis, orthoptic assessment and optometric assessment. It is also important to consider the use of questionnaires to assess traits such as quality of life, which can be markedly underestimated [27]. Examples of questionnaires used include the GO-QOL [28] and TED-QOL [29].

### Treatment of TED

Treatment should be multidisciplinary with the ophthalmologist, endocrinologist, radiologist, optometrist, orthoptist and general physician (GP). The principal aim should be thyroid function control as this is associated with a reduction in the severity of TED. General supportive measures should be immediately implemented or considered including the use of ocular lubricants, head elevation (gravity supports lid closure), taping lids closed at night, prisms in spectacles (to control diplopia), tinted spectacles (to hide eye appearance), counselling and support groups.

In the active phase of TED, systemic corticosteroids are considered and are most effective early in this phase, which prevents most of the morbidity associated with the disease. Other agents including ciclosporin, azathioprine, methotrexate, infliximab and rituximab are gaining popularity and some are the subject of clinical trials [14]. Selenium supplementation may also be used as it has been shown to significantly improve quality of life, reduce ocular involvement, and slow progression in patients with mild Graves' orbitopathy [30]. Orbital radiotherapy can be used as an adjunctive therapy and is particularly effective at improving ocular motility during active disease, due to the sensitivity of orbital lymphocytes to radiotherapy [31]. Rarely, surgical orbital decompression is required in cases of acute progressive optic neuropathy or exposure keratopathy.

Surgery for the improvement of cosmetic appearance and symptoms (e.g. diplopia) should be avoided if possible until the inactive phase (end of Rundle’s curve). The surgical options include decompression, motility and lid surgery (in that order). Decompression aims to improve proptosis and lid position. Motility surgery involves extraocular muscle repositioning to reduce or eliminate diplopia and/or abnormal head postures. Finally, lid surgery is used to reposition the lid(s).

### Treatment of hyperthyroidism

There are three main options in the treatment of hyperthyroidism: medical (carbimazole and propylthiouracil), radioactive iodine and surgery (ablation and thyroidectomy). Medical treatment blocks the production of thyroid hormones and is used until the patient is euthyroid. The drug is then tapered whilst maintaining normal free T4 levels. Alternatively a ‘block and replace’ regime may be used where higher drug doses are used along with thyroxine replacement. The major risk of this treatment is agranulocytosis. Radioactive iodine (single dose) is an effective alternative option but subsequent hypothyroidism is common. However, this treatment can cause progression of TED by a leakage of antigens from the thyroid gland, again eliciting an autoimmune response [3]. This risk can be reduced with the simultaneous use of corticosteroids [32]. Surgery is less commonly performed but may be used for patients with a large goitre.

## Conclusion

TED is a self-limiting orbital inflammatory condition with an active (inflammatory) and inactive phase. Risk factors include female gender, middle age and smoking. The majority of patients with TED have hyperthyroidism with the most common cause being Graves’ disease. There are numerous ophthalmic features; the two most serious being optic neuropathy and exposure keratopathy. Investigations include thyroid function, thyroid auto-antibodies, orbital imaging, visual field analysis, orthoptic assessment, and optometric assessment. There are a number of grading systems available to assess severity and to monitor activity of the disease. Management should be multidisciplinary with the ophthalmologist, endocrinologist, radiologist, optometrist, orthoptist and general practitioner.
